# Evaluation of the Performance of Helmet Prototypes Fabricated from Acrylonitrile Butadiene Styrene Composites Filled with Natural Resource

**DOI:** 10.3390/ma12010034

**Published:** 2018-12-22

**Authors:** Siti Nikmatin, Bambang Hermawan, Irmansyah Irmansyah, Mohammad Nur Indro, Ahmad Beng Hong Kueh, Achmad Syafiuddin

**Affiliations:** 1 Department of Physics, Faculty of Mathematics and Natural Sciences, Bogor Agricultural University, 16680 Bogor, Indonesia; bhermawan1000@gmail.com (B.H.); irmansyah@ipb.ac.id (I.I.); mnindro@gmail.com (M.N.I.); 2 Construction Research Centre (CRC), Institute for Smart Infrastructure and Innovative Construction (ISIIC), Faculty of Engineering, Universiti Teknologi Malaysia, 81310 UTM Johor Bahru, Johor, Malaysia; kbhahmad@utm.my; 3 Department of Civil Engineering, Faculty of Engineering, Universiti Malaysia Sarawak, 94300 Kota Samarahan, Sarawak, Malaysia; 4 Department of Water and Environmental Engineering, Faculty of Engineering, Universiti Teknologi Malaysia, 81310 UTM Johor Bahru, Johor, Malaysia; udenfisika@gmail.com

**Keywords:** oil palm, acrylonitrile butadiene styrene, natural fillers, mechanical properties

## Abstract

The performance of helmet prototypes fabricated from acrylonitrile butadiene styrene composites filled with oil palm empty fruit bunch fibers was evaluated. The fibers were produced using a milling procedure, while the composites were fabricated using a single-screw extrusion. The physical characteristics of the produced fibers, which are water content, size, and density, were investigated. In addition, the mechanical properties of the produced helmets, including shock absorption, yield stress, frequency, and head injury criterion (HIC), were examined. The impact strength of the produced helmets increases with the rise of filler content. In addition, the helmets were also able to withstand a considerable pressure such that the transmitted pressure was far under the maximum value acceptable by the human skull. The present work also found that HICs exhibited by the investigated helmet prototypes fulfill all the practical guidelines as permitted by the Indonesian government. In terms of novelty, such innovation can be considered the first invention in Indonesia since the endorsement of the use of motorcycle helmets.

## 1. Introduction

Helmets have been widely worn as a form of protective headgear, with the primary goal of reducing the risk of injury caused by impact incidents [1,2,3,4,5,6]. Although the development of helmets is well-established, there is a motivated necessity for fabricating helmet shells that meet certain stringent safety requirements, especially with respect to improving their energy absorption. In particular, the capability of motorcycle safety helmets to absorb the impact energy is one of the critical factors for consideration to reduce severe injury. A well-performing helmet must concurrently protect the human skull and brain, since impact damage induced during a motorcycle accident can cause death. Realizing the life-threatening risk of such an event, the understanding of skull fractures and brain damage inflicted during accidents, including their protection measure, have been of utmost priority to fabricators.

Several investigations have been carried out to study issues concerning head injury worldwide [7,8,9,10,11]. An investigation using 61 real-world accidents to evaluate the head injury criterion (HIC) and the head impact power (HIP), as well as the injury mechanisms, have been carried out [10]. These studies found that the moderate and severe neurological injuries cannot be distinguished solely on the global head acceleration, instead requiring a criterion computed using the intracranial variables. An experimental verification to identify the resonance frequencies of two kinds of freely vibrating human dry skulls and extrapolation of the results to living skulls by taking into account all known and estimated differences in mechanical properties has also been conducted [8]. Alternatively, a computational model employing the finite element model (FEM) to simulate the head injury was also proposed [7]. From these analyses, it was found that the composite shell systems exhibit lower shear performance, provide additional energy absorbing mechanisms, and result in better helmet crashworthiness behavior [9].

A good strategy to reduce the head injury during an accident is by involving a design with reputable criteria, as permitted by regulations given by a standardization. In order to improve the performance of helmets in terms of their energy absorption, recommendations for future helmets include the employment of materials that are capable of absorbing the impact energy during an accident, while keeping the acceleration transmitted to the head at a safe level. Therefore, it is constantly a great challenge to provide the right composition of helmet materials that have the proper set of mechanical properties. Several studies have reported that wearing helmets reduces fatalities by more than 25% [12,13]. Moreover, it has been proven that non-helmeted motorcyclists are up to 3.4-fold more likely to die compared to helmeted riders in traffic crashes [14].

Recently, the exploration and utilization of natural materials have formed huge engineering and commercial interests [15,16,17,18,19]. This is because the use of natural composite materials provides many benefits, encompassing not only the environment but also economy and social aspects [20]. Along with cost-saving and ecological benefits such as improvement in CO_2_-balance, the main motivation driving these developments is related to the mechanical property profiles of natural materials, which offer reinforcement potential [21]. Furthermore, several studies have proven that the performance of some natural materials in fiber form is closely comparable to those of synthetic glass fibers [22]. Combined with the low-density of natural fillers, employing natural fiber composites results in lighter structures when compared to mineral-, short glass fiber-, long glass fiber-, and short carbon fiber-reinforced materials [23]. In addition, natural fiber composites can also be processed similarly to these different composite classes, such as by employing injection molding and extrusion techniques. In terms of processing, it was well established that natural fiber composites offer advantages in regard to equipment wear [23].

Considering the aforementioned concerns, the present work aims to evaluate the performance of helmet prototypes produced from acrylonitrile butadiene styrene composites filled with oil palm empty fruit bunch fibers. Up to this instance, the composite formed by this type of natural fiber has not been examined as a helmet material. The present study offers an investigation into the advantage of natural resources as fillers for advanced engineering applications. The present study reveals that the proposed helmet prototype is in general suitable for applications, as regulated by the Indonesian National Standardization (SNI). It is worth noting that such innovation can be considered the first invention in Indonesia since the introduction of the use of motorcycle helmets. The first Indonesian mandatory helmet law was enacted in 1984. In 1986, the Department of Transportation instituted a regulation making helmet usage mandatory for all motorcyclists. This development is expected to contribute to reducing head and neck injuries and deaths from motorcycle crashes.

## 2. Materials and Methods

### 2.1. Materials

Oil palm empty fruit bunches (OPEFB) were collected from the PT Perkebunan Nusantara VIII Cikasungka, Bogor, Indonesia. Recycled and virgin acrylonitrile butadiene styrene (Torray Toyulac Resin 100MPJ40049689 NLG) were purchased from the PT MUB Jaya Cibinong, Bogor, Indonesia. A coupling agent, maleic anhydride, from Merck, Darmstadt, Germany was used in this work. In addition, an additive, antioxidant primer (butylated hydroxytoluene) from Tedia, Mumbai, India, was employed. The carvine 0331 Polyurethane 2K Z-331-039, carvine 0331 Polyurethane H-331-014, and carvine 0331.T-0378 were used for painting in three stages, namely, as the base, hardener, and thinner, respectively. The paints were supplied by the PT Murni Cahaya Pratama, Bogor, Indonesia. In addition, a polystyrene foam having a density of 33 g/cm^3^, wrapped using fabric with a thickness of 10 mm, and a visor fabricated using the polycarbonate were used to complete the overall structure of the helmet prototypes. 

### 2.2. Short Fibers Production

The OPEFBs were initially washed using tap water to remove any impurities, and then immersed in tap water for 72 h. To remove water content, the OPEFBs were dried under the sun for 24 h, followed by oven (Xenaco, Guangzhou, China) drying at a temperature of 100 °C for 8 h. The drying process was carried out such that the water content in the OPEFBs was less than 10%. Short fiber filler production was carried out using a hammer mill (Model HMV-4W-5.5, PT MUB Jaya Cibinong, Bogor, Indonesia) with a rate of 5000 rpm for 10 min. Hammer milling is a mechanical treatment to minimize particle size with the combined actions of collision, shaking, and milling.

### 2.3. Granular Production

Composition of composites for the granular production for all helmet samples is listed in Table 1. It is noted that SN1, SN2, and SN3 refer to the helmets with filler content of 15% and recycled acrylonitrile butadiene styrene (ABS), filler content of 15% and virgin ABS, and filler content of 20% and recycled ABS, respectively. The granular composite was produced using the single-screw extruder machine (Model HXSJ-125/125, Kaixin, Nanjing, China). In the preparation, the filler, ABS, coupling agent, and additive were mixed at a speed of 15,000 rpm for 15 min. The total mass of the sample was averagely measured as 50 kg. The mixed samples were processed using the single-screw extruder machine and blended with gradient temperatures of 195, 215, 220, 220, 220, 225, 225, and 225 °C.

In this process, composites having a granular diameter of 3 mm were obtained. In addition, this process can produce composites having a water content of about 30%. Next, the composites were dried under the sun for 24 h to reduce water content down to 13%, followed by oven drying at a temperature of 80 °C for 3 h to have a further water content reduction down to 7%. It is recommended that the water content of composites for helmet production using the molding injection machine is kept to be less than 10%.

### 2.4. Helmet Production

**Figure 1:**
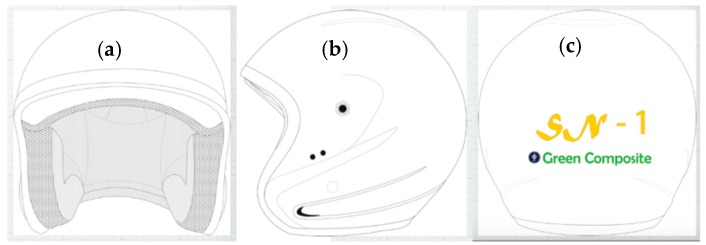
Design of the presently proposed helmet (**a**) front; (**b**) side and (**c**) back views.

Helmet prototypes were then fabricated using an injection molding machine (Model HC-250, Hwa Chin, Tainan, China). In the barrel, the samples were blended with gradient temperatures of 195, 215, 220, 220, 220, 225, 225, and 225 °C. In addition, the helmets were painted in three stages, namely, for the base, hardener, and thinner. In addition, the polystyrene foam and visor were installed in all the helmets. In general, the helmets were produced according to the National Indonesia Standardization (SNI). For demonstration, various viewing perspectives for the presently fabricated helmet are shown in Figure 1.

### 2.5. Shock Absorption Test

A shock absorption test was performed using the uniaxial impact machine (CADEX, model 1000_00_MIMA, Cadex Inc., Saint-Jean-sur-Richelieu, QC, Canada). This test was carried out according to the Indonesian National Standardization (SNI 1811-2007). The test was performed in three repetitions. Two different anvil types were employed for the test, i.e., hemispherical and flat. The hemispherical anvil has a spherical surface radius of 48 mm, constituting one-half of the surface of a full sphere. The flat anvil has a flat surface of minimum side dimensions of 125 mm and a thickness of 24 mm. Additionally, Flat-1 and Flat-2 anvils refer to the anvil dropped from 5 m and 3 m in height, respectively.

### 2.6. Yield Stress Estimation

The yield stress was estimated using the procedure proposed by Mills and Gilchrist [24]. It can be computed by considering the contact geometry between a flat impactor and the spherical outer surface of the foam liner. If the amount of liner crush, *x*, is less than the radius of curvature, *R*, of the spherical outer surface, the contacted area, *A*, can be estimated using *A* = 2*πRx*. If the impact is applied to a hemispherical anvil, the equation must be modified as Equation 1:

(1) $$A=2\pi x{\left(\frac{1}{R_{1}}+\frac{1}{R_{2}}\right)}^{-1}$$

where *R_1_* and *R_2_* are the radii of the helmet and anvil, respectively. It has been assumed that the foam yields over an area, *A*, of radius, *a*, with a constant yield stress, *σ*. The force transmitted by the foam can be estimated by *F* = *Aσ*. Therefore, the yield stress can be estimated using the following equation:

(2) $$\sigma =\frac{ma}{A}$$

where *m* is the mass of the helmet and head dummy and *a* is the acceleration of the helmet and anvil.

### 2.7. Frequency Analysis

In this analysis, a mass-spring-mass model developed by Gao and Wampler [25] was adopted. In general, it begins by applying Newton’s second law of motion and Hooke’s law formulas as:

(3) ma=−kx

By defining a function of the position of mass with respect to time as *x*(*t*) *= Acos*(*ωt*), Equation (3) becomes:

(4) $$\left(\frac{k}{m}-\omega^{2}\right)\left(Acos\left(\omega t\right)\right)=0$$

It can be obtained from Equation 4 that $\omega =\sqrt{k/m}$. Since *ω* = 2*πf*, the frequency, *f*, can be estimated using the following equation:

(5) $$f=\frac{1}{2\pi }\sqrt{\frac{k^{*}}{m}}\;\mathrm{with}\;k^{*}=\frac{k_{1}k_{2}}{k_{1}+k_{2}}$$

where *m* is the total mass of the helmet and head dummy and *k*_1_ and *k*_2_ are the stiffness constants of the helmet and head dummy, respectively.

### 2.8. Head Injury Criterion Analysis

A head injury can be defined as any incident that results in trauma to the skull or brain. Among all injury criteria, HIC is the most globally used for measuring the severity of injury in the cases where the human head is engaged as the impacted mass. By measuring the energy required to cause concussive effects, a limit between impact intensities causing fatal and non-fatal injuries can be determined. The analytical expression of HIC is described by the following equation [25]:

(6) $$\mathrm{HIC}={\left({\left[\frac{1}{t_{2}-t_{1}}\overset{t_{2}}{\underset{t_{1}}{\int }}\hat{a}\left(t\right)dt\right]}^{2.5}\left(t_{2}-t_{1}\right)\right)}_{\max}$$

where *t_1_* and *t_2_* are the initial and final times (in seconds) of the interval, during which HIC attains a maximum value, and acceleration, $\hat{a}$, is measured in gs (standard gravity acceleration). It is useful to note that the measurement in gs means that $\hat{a}$ is *a*/*g*, with *a* as the head acceleration and *g* as the acceleration of gravity in any compatible units. Therefore, $\hat{a}$ is defined as the normalized head acceleration. As an alternative, the overall normalized impact resistance index can be estimated for comparative purposes [26,27].

## 3. Results and Discussion

### 3.1. Physical Properties of the Produced Fibers

**Figure 2:**
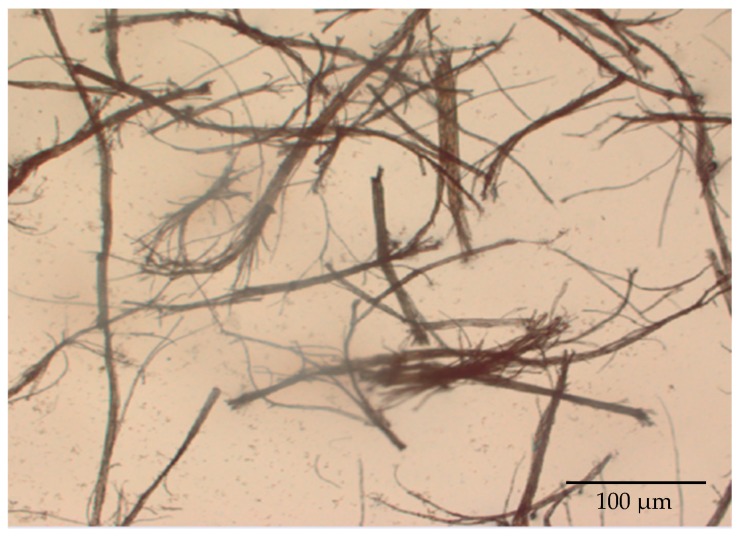
Morphology of the currently produced OPEFB.

The drying process carried out in this work is useful to ensure that the water content of the oil palm short fibers is less than 10%. They were 58.0%, 17.4%, and 7.2% for before drying, after drying under the sun, and after drying in an oven, respectively. It is noticeable that the lowest water content, which was less than 10%, can be obtained using the presently employed method. Figure 2 shows the morphology of the oil palm short fibers. The length and diameter of the short fibers were 230.1 ± 95.3 μm and 58.5 ± 23.0 μm, respectively. These values were obtained by the measurement of random samples using an optical microscopy.

Table 2 presents the densities of OPEFBs for different studies [28,29,30,31,32]. It is noticeable that the densities reported by various researchers are not identical because of the variation in the kind of oil palm fiber used. In addition, there exists some fluctuation in the irregular sectional areas, causing the length determination difference of OPEFBs [33]. Nevertheless, the current study found that the density of the produced fibers was in an acceptable range with those obtained from the previous works. Various investigations have reported that the densities of OPEFB fibers are in the range of 0.7 to 1.55 g/cm^3^, less than that of glass fiber, which is 2.6 g/cm^3^. This suggests that using OPEFB fibers as the reinforcement of a composite can reduce its total mass compared to the use of glass fiber. Therefore, studies on the exploration of OPEFB fibers as an alternative for glass fiber have been popular and of high potential for applications.

### 3.2. Helmet Prototype

**Figure 3:**
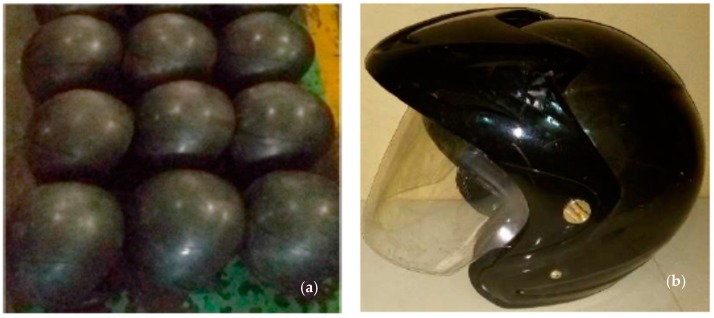
Presently proposed helmet prototype: (**a**) above and (**b**) side views.

Helmets produced from ABS composites filled with OPEFB fibers following the SNI 1811-2007 standardization can be well-expected to improve in their physical-mechanical properties, particularly in terms of good impact energy absorption during traffic accidents. Figure 3 shows the prototype of the presently produced helmet. In order to get an attractive appearance, the helmet was also painted. Helmet assembly was done by adding a layer of expanded polystyrene (EPS), soft lining, and other accessories required in accordance with SNI 1811-2007.

### 3.3. Mechanical Properties

Composite granules were used to produce the helmet specimens using the molding machine. Table 3 lists the impact characteristics of the helmets. As previously mentioned, all testing was conducted according to the SNI. The test was carried out at three different temperatures. This study found that the impacts of SN1 at a temperature of −20 °C were in the range of 81.3 to 121.7 G. In addition, their impacts at a temperature of 50 °C were in the range of 113.2 to 137.9 G.

As a comparison, the impact values for SN2 tested at temperatures of −20 °C and 50 °C ranged from 86.1 to 151.7 G and 101.7 to 130.3 G, respectively. In addition, the corresponding values for SN3 were 310.2 to 357.2 G and 230.1 to 270.5 G, respectively. It was found that the impact values for SN3 were higher compared to SN1 and SN2. Increase in the impact strength was possibly due to the increase in the filler content for SN3 (20%) compared to SN1 (15%) and SN2 (15%). Findings of this study are similar with those obtained from the previous works. For instance, increasing filler contents (snail shell powder) from 0% to 40% increased the impact strength of the produced composites [34]. 

A similar observation was also obtained when the composite material of low-density polyethylene (LDPE) as the base was mixed with raw kaolin [35]. From the relevant study, it was found that increasing the filler content from 5% to 15% increases their composite strength from 0.3 to 0.5 kJ/m^2^. An increase in the impact strength can be attributed to the elastic behavior of the added filler contents, which have high toughness and extendibility without a permanent deformation [36]. Moreover, the current study has confirmed that the produced helmets offer good performance according to the SNI in terms of their impact strength performance. 

### 3.4. Yield Stress and Frequency

The stress yields for SN1 were found to be in the range of 0.99 to 1.32 MPa and 25.94 to 40.65 MPa when the produced helmets were tested at temperatures of −20 °C and 50 °C using the flat and hemispherical anvils, respectively. At −20 °C, the frequency range was 50.74 to 94.21 Hz, while it was from 53.04 to 81.99 Hz at 50 °C. In summary, the yield stress and frequency characteristics of SN2 and SN3 are presented in Table 4 (detailed computation is provided in the supporting document, A1).

### 3.5. HIC Characteristics

Based on ASTM-F1292-04, the time interval for evaluation for impact performance is restricted to a maximum of 15 ms, and HIC < 1000 is known as a critical value for avoiding fatal injuries to the human head. This implies that a very high head acceleration is tolerable for a brief period of time. The probabilities of brain injury at different HIC scores are documented in the ASTM-F1292-04 standardization as listed in Table 5. The values shown emphasize the importance of proper and effective protection.

**Figure 4:**
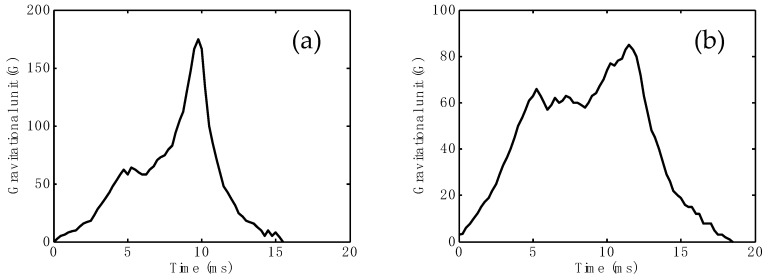
Typical head injury criterion curves in accordance with SN1, with testing conditions of (**a**) −20 °C and (**b**) 50 °C. Other data for SN2 and SN3 are provided in the supporting document, A1.

HIC scores of the presently proposed helmet are presented in Table 6. In addition, HIC curve examples of the prototype helmet with testing temperatures of −20 °C and 50 °C are shown in Figure 4. HIC scores were found in the range of 804.69 to 828.38 when the helmets were tested at −20 °C. At 50 °C, the HICs were in the range of 769.63 to 792.75. Based on the HIC listed in the table, it is ratified that the presently proposed helmet can be categorized as offering good protection, with a 95% chance of minor injury, 70% chance of moderate injury, and 4% chance of critical injury. For a comprehensive overview, HIC characteristics of SN2 and SN3 are also presented in Table 6. Therefore, it is obvious that the proposed helmet has a great conformity with the Indonesian standard, SNI 1811-2007. This is because SNI 1811-2007 has regulated that the permitted HIC is <3000. 

## 4. Conclusions

This study was carried out to evaluate the performance of helmet prototypes produced from acrylonitrile butadiene styrene composites filled with oil palm empty fruit bunch fibers. The mechanical properties of constituents for the helmet prototype have been characterized and presented. The presently fabricated fiber has a water content of less than 10%, density of 1.35 g/cm^3^, fiber length of 230.1 ± 95.3 μm, and fiber diameter of 58.5 ± 23.0 μm. The impact values for SN3 were the highest compared to SN1 and SN2, suggesting an improved impact strength for a higher filler content. Furthermore, it was also found that all HICs produced by the helmet prototypes were <850, as permitted by the SNI regulation. In closing, the prototype helmets are in practical agreement and conform to the Indonesian standard, SNI 1811-2007. 

## Figures and Tables

**Table 1:** Material composition of the presently proposed helmets.

Type of Helmet	Filler (%)	Type of ABS	Coupling Agent (%)	Additive (%)
SN1	15	Recycle	2	1
SN2	15	Virgin	2	1
SN3	20	Recycle	2	1

**Table 2:** Densities of the studied filler from various works.

Type of Oil Palm Fiber	Density (g/cm^3^)	Country	Reference
Short fibers	0.7 to 1.55	India	Sreekala et al. [31]
Short fibers	1.03	India	Rao and Rao [30]
Short fibers	1.4	India	Joseph et al. [28]
Short fibers	1.03	India	Rao and Rao [30]
Short fibers	1.15	Malaysia	Yusoff et al. [32]
Short fibers	1.15	Indonesia	Karina et al. [29]
Short fibers	1.35	Indonesia	Present work

**Table 3:** Impact characteristics of the currently produced helmets.

Code	Temperature (°C)	Clash Position	Anvil Type	Impact (G)
SN1	−20	Backside	Flat-1	121.7
			Flat-2	103.6
		Topside	Hemispherical	81.3
	50	Backside	Flat-1	137.9
			Flat-2	113.2
		Topside	Hemispherical	127.4
SN2	−20	Backside	Flat-1	151.7
			Flat-2	133.6
		Topside	Hemispherical	86.1
	50	Backside	Flat-1	130.3
			Flat-2	122.2
		Topside	Hemispherical	101.7
SN3	−20	Backside	Flat-1	317.1
			Flat-2	357.2
		Topside	Hemispherical	310.2
	50	Backside	Flat-1	240.5
			Flat-2	270.5
		Topside	Hemispherical	230.1

**Table 4:** Yield stress and frequency of the tested helmets.

Code	Temperature (°C)	Clash Position	Anvil Type	Yield Stress (MPa)	Frequency (Hz)
SN1	−20	Backside	Flat-1	1.17	54.99
			Flat-2	0.99	50.74
		Topside	Hemispherical	25.94	44.95
	50	Backside	Flat-1	1.32	58.54
			Flat-2	1.08	53.04
		Topside	Hemispherical	40.65	56.26
SN2	−20	Backside	Flat-1	1.45	61.4
			Flat-2	1.28	57.62
		Topside	Hemispherical	27.48	46.25
	50	Backside	Flat-1	1.25	56.9
			Flat-2	1.17	55.1
		Topside	Hemispherical	32.45	50.27
SN3	−20	Backside	Flat-1	3.04	88.77
			Flat-2	3.42	94.21
		Topside	Hemispherical	98.99	87.80
	50	Backside	Flat-1	2.30	77.31
			Flat-2	2.59	81.99
		Topside	Hemispherical	73.43	75.62

**Table 5:** Head injury criteria.

HIC Score	Moderate Injury (%)	Moderate Injury (%)	Critical Injury (%)	Fatal (%)
0	0	0	0	0
250	40	20	0	0
500	80	40	2	0
750	95	70	4	0
1000	98	90	8	2
1250	100	95	10	2
1500	100	98	20	4
1750	100	100	45	10
2000	100	100	70	30
2250	100	100	90	70
2500	100	100	95	90
2750	100	100	98	95
3000	100	100	100	100

**Table 6:** Head injury criteria of the presently proposed helmets.

Code	Temperature (°C)	Clash Position	Anvil Type	HIC
SN1	−20	Backside	Flat-1	760
			Flat-2	398
		Topside	Hemispherical	335
	50	Backside	Flat-1	799
			Flat-2	433
		Topside	Hemispherical	453
SN2	−20	Backside	Flat-1	1141
			Flat-2	662
		Topside	Hemispherical	334
	50	Backside	Flat-1	980
			Flat-2	587
		Topside	Hemispherical	411
SN3	−20	Backside	Flat-1	904
			Flat-2	858
		Topside	Hemispherical	694
	50	Backside	Flat-1	591
			Flat-2	595
		Topside	Hemispherical	603

## Supplementary Materials

The following are available online at http://www.mdpi.com/1996-1944/12/1/34/s1.
